# Therapeutic Potential of Annexin A1 in Ischemia Reperfusion Injury

**DOI:** 10.3390/ijms19041211

**Published:** 2018-04-16

**Authors:** Junaid Ansari, Gaganpreet Kaur, Felicity N. E. Gavins

**Affiliations:** 1 Department of Molecular & Cellular Physiology, Louisiana State University Health Sciences Center-Shreveport, Shreveport, LA 71130, USA; jansar@lsuhsc.edu (J.A.); gkaur3@lsuhsc.edu (G.K.); 2 Department of Neurology, Louisiana State University Health Sciences Center-Shreveport, Shreveport, LA 71130, USA

**Keywords:** ischemia–reperfusion injury, Annexin A1, formyl peptide receptors, ischemic stroke

## Abstract

Cardiovascular disease (CVD) continues to be the leading cause of death in the world. Increased inflammation and an enhanced thrombotic milieu represent two major complications of CVD, which can culminate into an ischemic event. Treatment for these life-threatening complications remains reperfusion and restoration of blood flow. However, reperfusion strategies may result in ischemia–reperfusion injury (I/RI) secondary to various cardiovascular pathologies, including myocardial infarction and stroke, by furthering the inflammatory and thrombotic responses and delivering inflammatory mediators to the affected tissue. Annexin A1 (AnxA1) and its mimetic peptides are endogenous anti-inflammatory and pro-resolving mediators, known to have significant effects in resolving inflammation in a variety of disease models. Mounting evidence suggests that AnxA1, which interacts with the formyl peptide receptor (FPR) family, may have a significant role in mitigating I/RI associated complications. In this review article, we focus on how AnxA1 plays a protective role in the I/R based vascular pathologies.

## 1. Introduction

Cardiovascular diseases (CVD) are the main cause of morbidity and mortality worldwide and were solely responsible for the death of about 17.7 million people in 2015, with the vast majority being due to either ischemic heart disease (IHD) or cerebrovascular disease [1]. This has led to the exponential increase in healthcare costs with estimated increase in total medical care costs in United States (US) alone rising from $396 billion to $918 billion between the years 2012 and 2030. Additionally, the burden of CVDs has increased tremendously in developing countries and are predicted to overtake infectious disease as the leading cause of mortality by the year 2020 [2].

Many acute cardiovascular events, such as acute ischemic stroke (AIS) and myocardial infarction (MI), are due to, e.g., unhealthy vascular states, resulting from prolonged atherosclerosis, coronary artery disease, diabetes, uncontrolled hypertension, and aging [1]. Additional studies have suggested behavioral, psychosocial including bereavement [3] and environmental factors can also trigger and contribute to acute cardiovascular events [4].

Both AIS and MI are life-threatening conditions which require prompt treatment consisting of rapid restoration of blood flow followed by subsequent management consisting of anti-inflammatory strategies and neuro- and cardio-protection for secondary prevention [5,6]. However, restoration of blood supply and re-oxygenation paradoxically lead to further exaggeration of tissue injuries and tissue destruction (termed ischemia–reperfusion injury (I/RI)). To restore homeostasis, it is essential that the ensuing inflammatory and thrombotic environment is resolved. Resolution involves a tightly orchestrated series of active events and is now accepted as one of the four major outcomes for acute inflammation, the others being progression to chronic inflammation, scarring, and fibrosis [7,8,9]. Several anti-inflammatory mediators which have pro-resolving properties have been proposed over the years, such as resolvin D1 (RvD1), RvD2, RvE1, maresins, protectin D1 and lipoxin A_4_ (LXA_4_) [10,11]. Another known anti-inflammatory and pro-resolving mediator, which is the focus of this review, is the glucocorticoid-regulated protein Annexin A1 (AnxA1). AnxA1 has a particular appeal for drug discovery programs as it is founded on endogenous anti-inflammatory molecular pathways and as such is attractive because it can mimic the body’s own pro-resolution effects while potentially having fewer side effects than existing therapeutic agents [7]. This review aims to highlight the issues associated with I/RI. We also focus on AnxA1, its history, mechanism of action and its role in I/RI in different organs including brain, heart, kidney and gastrointestinal tract. Finally, we discuss the potential of AnxA1 in drug discovery programs for the treatment of I/RI.

## 2. Ischemia Reperfusion Injury (I/RI)

I/RI is a fundamental vascular pathobiological paradigm that contributes to the pathophysiology of various acute CVDs such as IHD and AIS, along with other pathophysiological responses such as transplantation and acute kidney injury (AKI). I/RI occurs in two distinct phases: the initial ischemic insult caused by vascular occlusion, and the subsequent reperfusion phase manifested by a profound local and systemic inflammatory response (called “reperfusion injury”) [12,13]. The term “ischemia” refers to deficient blood supply to tissues due to obstruction of the arterial inflow. The severity of cell dysfunction is influenced by the extent and duration of obstruction. Treatment for ischemic events is always restoration of blood flow to the affected organ at the earliest time possible (with some organs such as the brain being far more susceptible to damage: “time is brain”). However, reperfusion itself can induce and exaggerate tissue injury because it results in a gamut of inflammatory mediators including reactive oxygen species (ROS), increased cell adhesion molecules (e.g., intracellular adhesion molecule-1 (ICAM-1)), heightened production of lipid mediators including leukotriene B_4_ (LTB_4_) and platelet activating factor (PAF) [14].

A wide range of pathological processes contribute to I/R-associated tissue damage. Prolonged occlusion triggers depletion of adenosine triphosphate (ATP) and decreases the intracellular pH as a result of anaerobic metabolism and lactate accumulation [13]. ATP depletion inactivates ATPases (e.g., Na^+^/K^+^ ATPase), reduces active calcium ion (Ca^2+^) efflux and limits the reuptake of Ca^2+^ by the endoplasmic reticulum (ER), thereby producing calcium overload in the cell. These changes are accompanied by opening of the mitochondrial permeability transition (MPT) pore, which dissipates the mitochondrial membrane potential and further impedes ATP production. Depletion of ATP also leads to accumulation of xanthine oxidase (XO) which is formed from xanthine dehydrogenase (XD) under hypoxic conditions, allowing for a burst of superoxide and hydrogen peroxide production and initiating the overall I/RI [15]. The re-oxygenation in reperfusion phase enhance the production ROS. During ischemic phase, electron-transport enzymes, including mitochondrial respiratory chain, are damaged. Therefore, incomplete oxidation of molecular oxygen occurs leading to generation of more ROS and exaggerating tissue injury [16].

An immune response elicits upon reperfusion and consists of both innate and adaptive immune systems. The innate immune response involves signaling events through pattern recognition molecules (especially toll like receptors (TLRs)). Ligands such as components of damaged tissue binding to TLRs lead to the activation of downstream signaling pathways, including NF-kappa-B (NF-κB), mitogen activated protein kinase (MAPK) and type I interferon pathways, resulting in the induction of pro-inflammatory cytokines and chemokines [17]. Upon reperfusion, the ischemic area acquires multiple activated leukocyte types (e.g., neutrophils, monocytes, and lymphocytes), dominated initially by neutrophil infiltration. I/RI also activates antigen-specific T cells by mechanisms that are not yet well characterized. Recent evidence suggests both antigen-specific and independent mechanisms of activation where T cells accumulate at sites of ischemia and reperfusion [18,19]. Multiple studies have shown that enhanced generation of oxidants results in the activation and deposition of complement and phospholipase A_2_ (PLA_2_)-mediated production of LTB_4_ and platelet activating factor (PAF). Furthermore, there is abundant evidence suggesting the role of platelets in I/RI [20] by mediating microvascular thrombosis as well as ensuing inflammation through platelet–platelet as well as platelet–leukocyte interactions. These communications lead to the release of a broad range of pro-inflammatory molecules including high-mobility group box 1 protein (HMGB1), cell differentiation 40 ligand (CD40L), PolyP, and interleukin-1 alpha/beta (IL-1α/β) furthering the thrombo-inflammatory environment and contributing in both innate and adaptive immune responses [21].

## 3. Annexin A1

Annexin A1 (AnxA1) is a 37-kDa member of superfamily of thirteen annexin proteins, comprising 346 amino acids with at least twelve distinct Ca^2+^ and phospholipid binding proteins [7]. This member of the superfamily was aptly named AnxA1 due to its capability to “annex” phospholipid membranes [7]. The protein itself was discovered in the 1970s and 1980s by its ability to inhibit PLA_2_ activity and eicosanoid synthesis [7]. Further anti-inflammatory actions were seen in guinea pig lung models, glucocorticoid (GC)-treated rat models and rat medulla cell experiments [7]. AnxA1 has a unique N-terminal region (active biological portion) embedded within its core, which is only exposed under elevated Ca^2+^ levels (≥1 mM). The N-terminal region of each member of the annexin superfamily is believed to be the “fingerprint”, endowing its biological activity, however there are other regions of interest in the core protein which are known to also possess anti-inflammatory properties, for instance, Antifammin-2 [7,22].

AnxA1 is abundantly present in cells of myeloid origin such as neutrophils, monocytes and macrophages. Under resting conditions, AnxA1 is present within the cytoplasm and is only extruded upon cell activation [7]. The molecular mechanisms responsible for secretion vary from cell to cell, e.g., in neutrophils, AnxA1 resides in the gelatinase granules and is released upon neutrophil activation (e.g., neutrophil–endothelial interaction), attaching itself to the outer leaflet of the plasma membrane and undergoing further conformational change to expose the N-terminal region [7]. In the case of macrophages, AnxA1 is released via the ATP-binding cassette (ABC) transporter system [7]. AnxA1 is regulated by GCs, with GCs increasing both the *AnxA1* gene and also its secretion from existing intracellular pools by stimulating protein kinase C (PKC) activity [7].

The anti-inflammatory and pro-resolving effects of this 37 kDa protein are mediated by the shedding of l-selectin resulting in decreased neutrophil adhesion to the endothelium and restricting transmigration [7]. Pharmacological intervention of AnxA1 has been shown to decrease neutrophil rolling and adhesion to endothelium while increasing detachment of adherent and inducing neutrophil apoptosis [23]. Perretti and Flower (1993) demonstrated that AnxA1 attenuated IL-1 and IL-8 induced neutrophil migration into the murine air pouch [24]. Additionally, Getting et al. showed that both endogenous and exogenous AnxA1 were able to inhibit the neutrophil and monocyte recruitment in murine peritoneal cavity [25]. These findings suggest that AnxA1 retains its anti-inflammatory action irrespective of stimuli. Furthermore, AnxA1 may further undergo changes such as cleavage, induced by neutrophil specific proteases (e.g., Cathepsin G (CG) [26], Neutrophil elastase (NE) and Proteinase-3 [27]), although it is unknown as to whether the cleavage process occurs to inactivate AnxA1 and produce homeostasis, or to produce bioactive fragments which can act as a pro-drug [7].

### AnxA1 and the Formyl Peptide Receptors (FPRs)

AnxA1 and its mimetic peptides, such as the N-terminal derived Ac2-26, bind to the formyl peptide receptor (FPR) family of seven transmembrane G-protein-coupled receptors (GPCRs) [28]. Various cell types express FPRs, especially myeloid cells, e.g., neutrophils and monocytes. Three FPR members exist in humans and they are termed: FPR1, FPR2/ALXR (also known as the LXA_4_ receptor), and FPR3. FPR2/ALXR shares 69% amino acid sequence homology with FPR1, and FPR3 shares 56% amino acid homology to FPR1 and 72% to FPR2/ALX [7,29]. In mice, the FPR family is more complex, consisting of at least eight members. Mouse Fpr1 shares 77% sequence homology with human FPR1, and Fpr2 has 76% homology to FPR2/ALXR [29].

The FPRs are primarily coupled to G proteins (G_Iα2_, G_Iα3_). Upon the binding of a ligand, such as formyl-Met-Leu-Phe (fMLP), G proteins are activated and trigger the release of several second messengers such as Ca^2+^ from intracellular stores, through activation of phospholipase C (PLC), PLD and PLA_2_ [30]. Neutrophils predominantly sense inflammatory stimuli via FPRs to perform both pro- as well as anti-inflammatory functions, depending upon the pathophysiological status and ligand binding. In inflammatory states, neutrophil FPRs participate in various biological functions including chemotaxis, degranulation, ROS production, promoting neutrophil–platelet interactions [31], and enabling apoptosis and phagocytosis [32]. The ability of FPRs to perform such wide range of biological functions is due to their ability to interact with multitude of agonists and antagonists, ranging from formylated and non-formylated proteins/peptides to small molecular weight compounds, e.g., fMLP, His-Phe-Tyr-Leu-Pro-Met (HFYLPM) (chemoattractants for monocytes and neutrophils), AnxA1, and HIV envelope proteins gp41 and gp120 [30]. A detailed description of the FPRs, their ligands and biological functions is given in Table 1 and Table 2.

Among the FPR members, FPR2/ALX is the most versatile and can interact with a multitude of ligands resulting in both anti-inflammatory and pro-resolving (AnxA1 and LXA_4_) as well as pro-inflammatory (serum amyloid A (SAA) and cathelicidin) functions (Table 2). These diverse effects also seem to be partly due to the ability of FPR2/ALX to facilitate biased agonism and enable different dimerization states after ligand binding [32,33]. Binding of SAA and/or the cathelicidin-associated antimicrobial peptide leucine-37 (LL37) to FPR2/ALX results in neutrophil NF-κB activation, cytokine release, increased neutrophil infiltration and lifespan [32]. However, binding of anti-neuronal nuclear antibody (AnnA1) inhibits neutrophil infiltration, promotes neutrophil apoptosis, and pushes macrophages towards a less pro-inflammatory phenotype, increasing the rate of phagocytosis by macrophages [32]. Ongoing studies are showing that FPR1 signaling is associated with neutrophil oxidative burst by inducing a rapid and reversible increase in the mitochondrial membrane potential within neutrophils [32]. In addition, mitochondrial-derived FPR1 ligands also function as chemotactic damage-associated molecular pattern molecules (DAMPs also known as alarmins or danger signals), activating the innate immune system [34].

The function of FPR3 is less well characterized, although it is expressed on eosinophils, monocytes, macrophages, and dendritic cells. Evidence suggests that FPR3 plays role in allergic reaction and dendritic cell maturation [35].

## 4. Acute Ischemic Stroke (AIS)

Cerebrovascular disease is the second most common cause of death after IHD, the third leading cause of disability-adjusted life-years (DALYs) and accounted for eight million deaths worldwide in 2013 [1,5]. AIS is the most common manifestation of cerebrovascular disease with limited therapeutic options to date [5], mainly due to complex pathophysiology. AIS evolves locally due to underlying atherogenesis and vasculopathy (thrombotic occlusion), or from a distant embolus arising from the heart (embolic occlusion) [36]. These events result in activation of an ischemic cascade, culminating in sudden tissue hypoxia, cellular dysfunction, cell death and subsequent disruption of blood–brain barrier [37]. The main goal in AIS management is rapid recanalization of the occluded vessel to restore blood supply and limit focal ischemic insult and global hypoxic injury. However, various animal models have demonstrated that reperfusion can actually result in tissue injury at both molecular and cellular levels [37] as translated into recurrent strokes seen in many patients [38]. As such, there are several strategies currently under development to generate neuroprotective agents to prevent I/RI and irreversible brain injury in AIS [38].

I/RI plays a significant detrimental role in AIS resulting in widespread inflammation due to immune responses. Ischemia results in the complex accumulation of various cellular mediators including activated leukocytes (neutrophils, monocytes and lymphocytes), activated platelets, alongside activation of resident cells (e.g., microglia and astrocytes) superoxide generation and cytokines (tumor necrosis factor-α (TNF-α) and interleukin-1 beta (IL-1β)) production [39]. Under hypoxia, the deficiency of vasodilator in particular endothelial nitric oxide (NO) has been shown to enlarge stroke size [40]. TLRs have also been shown to play a significant role in cerebral I/RI especially mediating responses through microglial cells, and TLR_4_ has been demonstrated to upregulate the expression of cellular adhesion molecules (CAMs and selectins), facilitating neuronal injury [41]. The activation of microglia and macrophages increases the expression of, e.g., ICAM-1, P and E-selectin, which enhance leukocyte rolling and sticking to the vessel surfaces [40]. Additionally, there is a crosstalk between thrombosis and inflammation in cerebral ischemia resulting in a thrombo-inflammatory state [42]. Subsequently, restoration of blood flow results in cellular and molecular responses involving innate as well as adaptive arms of immunity, activation of the complement system, and the coagulation system. These responses lead to a further damage of the affected tissue. Additional injury is caused by the production of ROS, and the involvement of other inflammatory cells such as dendritic cells [37,43].

Neutrophils play a huge role as these are first immune cells to infiltrate the ischemic region of brain [44] and accumulate within six hours of reperfusion and increase the tissue infarct size between six and twenty-four hours (the time of maximal infarct related closely with the time course of neutrophil infiltration) [44]. During reperfusion, in addition to interacting with other cells, e.g., endothelial cells and platelets, neutrophils also produce many pro-thrombotic mediators such as neutrophils extracellular traps (NETs) and serine proteases, e.g., CG and NE, which promote thrombus formation. Furthermore, matrix metalloproteinase-9 (MMP-9) and the surface integrin receptor Mac-1 (CD11b/CD18 or macrophage-1 Ag), expressed by neutrophils play significant roles in compromising blood–brain barrier (BBB) and accelerating thrombus formation by binding to fibrinogen, von Willebrand factor (vWF), and glycoprotein Ib (GPIb), respectively [42,45].

### The Role of Annexin A1 in AIS

AnxA1 has been shown to act in various ways to mitigate cerebral I/RI. Our work has shown a significant effect of AnxA1 (and its peptide mimetics) on diminishing leukocyte adhesion and trafficking (mainly by acting in an in an autocrine/paracrine fashion to decrease leukocyte–endothelial interactions) [46], inhibiting the release of pro-inflammatory and pro-thrombotic cytokines, and regulating neutrophil–platelet interactions [7,31,47]. Human recombinant AnxA1 and its peptide mimetics have shown to markedly reduce the lesion size, clinical score and markers of leukocyte infiltration in murine middle cerebral artery occlusion (MCAo) model [31,46,47]. Recently, we showed that exogenous administration of AnxA1 N-terminal peptide Ac2-26 attenuated neutrophil and platelet activation and neutrophil–platelet aggregation in the murine cerebral microvasculature after induction of cerebral I/RI in mice [31]. Ac2-26 has also been shown to be protective in rats, as demonstrated by intracerebroventricular administration of the peptide reducing infarct size and cerebral edema two hours post cerebral ischemia [48]. McArthur et al. demonstrated that animals lacking the AnxA1 receptor Fpr2/ALX (i.e., Fpr2/ALX knock-out or Fpr2/ALX^−/−^ mice) showed markedly greater BBB leakage post-ischemia than their wild-type (WT) counterparts [49]. Additionally, AnxA1 plays a role in mediating the permeability of the BBB. Cristante et al. showed that AnxA1 knock-out (AnxA1^−/−^) mice possess a disorganized actin cytoskeleton due to increase in activity of RhoA small GTPase and a disruption of occludin and VE-Cadherin [50]. Furthermore, it is not just blood cells that express Fpr2/ALX, but also brain resident cells such as Microglia [51] and as such are potential targets for the anti-inflammatory and pro-resolving effects of Ac2-26 and its parent compound [30].

## 5. Ischemic Heart Disease (IHD)

IHD is the leading cause of mortality with an estimated 8.2 million deaths worldwide in 2013 [1]. Despite significant improvement over the years in survival rates (due to advances in reperfusion therapy), secondary heart failure, due to ensuing inflammation, is a major cause of disease burden after myocardial infarction. Overproduction of ROS [52], cardiac tissue remodeling and ensuing pro-inflammatory immune response are the main causes of MI/RI [33]. The pharmacological administration of anti-oxidants and calcium channel blockers has shown the promising results under experimental settings, however both interventions failed to achieve success in clinical studies [53].

If MI lasts more than twenty minutes, cardiomyocyte death is initiated in the subendocardium, expanding towards epicardium [53]. The depletion of ATP under ischemia due to halt in oxidative phosphorylation can inhibit myocardial contractile function [53]. Ischemia leads to Ca^2+^ overload, which can cause damage to ultrastructure of myocardial [54]. Initially, it was thought that reperfusion can limit infarct size and preserve left ventricular (LV) systolic function, however, with growing scientific evidence, it was proven that reperfusion can itself induce cardiomyocyte death [53]. Reperfusion in IHD results in a massive outpouring into the ischemic tissue of activated leukocytes (especially neutrophils) and other mediators e.g., complement fragments, ROS and pro-inflammatory cytokines, all of which promote further injury and cardiomyocyte death [33]. Several interventions have been shown to reduce infarct size up to 50% under experimental MI/R conditions. These interventions include administration of biologics to inhibit P-selectin, CD11, CD18 and ICAM-1 and pharmacologic inhibition of complement activation and leukocyte depletion [55].

Neutrophils play a central role in the MI/RI. They are the first cells to be summoned, resulting in accelerated pro-inflammatory responses including production of inflammatory mediators (e.g., CG, NE and MMP-9 [56]) and ROS, leading to microvascular injury, cardiomyocyte death and extracellular matrix (ECM) degradation and remodeling [57]. Neutrophil activation in I/RI promotes the expression of adhesion molecules, leading to adherence of neutrophils to the endothelium followed by transmigration, and direct interaction with the cardiac myocytes [57].

### The Involvement of AnxA1 in IHD

AnxA1 and its N-terminal peptides have both been shown to be cardioprotective in various I/RI models [58,59]. D’Amico et al. demonstrated a decrease in infarct size (50%) upon infusion of recombinant AnxA1 and the protective effects of AnxA1 were further confirmed by La et al. as evidenced by the administration of the AnxA1 N-terminal peptide, Ac2-26, to decrease infarct size and reduce MPO and IL-1β content in infarcted hearts [58]. Treatment with AnxA1 also attenuated loss of fiber organization, decreased MPO activity, reduced TNF-α and macrophage inflammatory protein (MIP-1α) levels and leukocyte extravagation in cardiac tissues [60]. Peptide Ac2-26 has been shown to preserve cardiomyocyte contractile function and reduce cardiac myocyte injury, with protection, at least in part, being associated with activation of PKC, p38-MAPK and K_ATP_ channels [61]. Additionally, Qin et al. demonstrated that exogenous administration of Ac2-26 at the onset of reperfusion increased cardiomyocyte viability and recovery of LV function, which was associated with FPR1/Akt signaling [62].

Hematopoietic progenitor stem cell (HPSC) mobilization and differentiation has been shown to be regulated by AnxA1 [63], e.g., AnxA1 deficiency results in increased cardiac necrosis, inflammation, hypertrophy and fibrosis resulting in exaggerated cardiac infarct size after eight days of myocardial infarction [63]. These effects were linked to a greater expansion and altered mobilization of HPSC, with increased circulating neutrophils and platelets and altered macrophage inflammatory phenotype and function [63].

More recently, there has been a large focus on the pharmacological concept of biased-agonism (multiple active conformations of a receptor exist and elicit distinct signals yielding to multiple functional outcomes). The small-molecule FPR1/FPR2/ALX agonist, Compound 17b (Cmpd17b), has been shown to have biased agonism, by enabling FPR based pro-resolving biology without changing FPR1/2-mediated calcium mobilization, hence providing superior cardio-protection. Cmpd17b, similar to AnxA1, attenuates both early and late inflammatory responses after reperfusion in MI [64], with ERK1/2–Akt kinases seeming to play a significant role [33,64].

## 6. I/RI in the Kidney

Kidney transplantation is the leading cause of kidney I/RI. At the end of 2016, there were approximately 100,000 people waiting for a kidney transplant. Due to greater availability, the vast majority of kidney transplants which take place use organ(s) from a cadaveric donor. For instance, out of 17,107 kidney transplants that took place in 2014, 11,570 organs came from cadaveric donors and only 5537 came from living donors [65]. However, cadaveric donor kidneys are more susceptible to I/RI due to prolonged ischemia under cold conditions [66]. In the case of living donors, although ischemic period is shorter, the organ is still subjected to warm ischemia during harvesting which may also result in I/RI [66]. Finally, a more severe injury occurs during the reperfusion phase in the recipient due to the activation of innate and adaptive immune responses. Indeed, many renal transplant recipients are under immunosuppressant therapy to prevent graft dysfunction. Figures show that 3% of kidney transplants will fail within a month of transplantation, 7% within a year and 17% within three years. Furthermore, each year, more than 20% of total kidney transplants are re-transplanted due to failure of the transplanted kidney [67]. Therefore, there is an urgent unmet clinical need to find better therapies to prevent graft rejection.

Renal I/RI is characterized by a decrease in glomerular filtration rate (GFR, which is the volume of the fluid filtered from the kidney each minute). Ischemia leads to the depletion of ATP which in turn inhibits the activity of tubular Na ^+^K^+^ ATPase and the redistribution of the Na^+/^H^+^ exchanger. Both conditions cause a decrease in the reabsorption of Na^+^ by tubular cells. Due to increased Na^+^ filtration, GFR is limited via tubuloglomerular feedback mechanism to avoid Na^+^ and water loss [68]. Additionally, ROS production is increased during I/RI causing renal tubule injury via oxidation of proteins and lipids on membrane, DNA damage and thereby triggering apoptosis of tubular epithelial cells which can shed into tubular lumen causing tubular obstruction [69,70]. The paracellular diffusion of water, ions and macromolecules becomes unregulated upon the loss of tubular cells and this increases the back-leak into the interstitium and blood vessels, which also decreases GFR. Thus, it can be said that GFR can also be decreased by the synergetic effect of cell surface area and tubular obstruction. Additionally, apoptosis leads to the decrease in cell–cell interactions increasing the vascular permeability [69,71]. The depletion of ATP also inhibits the activity of Ca^2+^ ATPase causing an increase in intracellular calcium levels which can induce cytoskeletal damage and increase in vascular permeability [69].

Inflammation plays a major role in renal I/RI due to enhanced production of pro-inflammatory cytokines, e.g., IL-6 and TNF-α, which have been shown to work mainly through JAK/STAT signaling pathways such that inhibition of the JAK/STAT signaling pathway has been shown to improve kidney I/RI outcomes. Other cytokines, e.g., MCP-1, IL-1β, IL-6, and IL-18, are released upon renal tubular cell activation and further the damage due to I/RI [72]. As with other I/RIs, in renal I/RI, neutrophils are the first cells to respond to the injury site, which results in the production of multiple mediators including NE, tissue type plasminogen activator, hepatocyte growth factor and CD44 expression. Neutrophils also cause renal microvasculature obstruction as they bind to endothelial cells and platelets. Additionally, adhesion molecules, e.g., ICAM-1 and P-selectin, which are major regulators of neutrophil recruitment have been shown to be involved in kidney I/RI. More recently, Kelly et al. showed that kidneys from ICAM-1^−/−^ mice displayed protection against I/RI, which was further confirmed by blocking ICAM-1 with an ICAM-1 specific antibody [73,74]. Takada et al. showed that soluble P-selectin ligand decreased the neutrophil transmigration in rat ischemic kidneys [75].

### The Association of AnxA1 in Renal I/RI

The role of AnxA1 in kidney I/RI is in its nascent stage. However, some studies have shown that AnxA1 may exert a protective role, e.g., in a rat model of renal I/RI, exogenous administration of peptide Ac2-26 reduced: (a) tubular necrosis; (b) the influx of phagocytic cells (neutrophil and monocytes); (c) sodium and potassium excretion; and (d) GFR, thereby improving functional recovery and outcome [76].

Cyclosporine is an immunosuppressant and is used to avoid organ transplant rejection. The major side effect of Cyclosporine treatment is nephrotoxicity, as the compound decreases renal blood flow and increases renal vascular resistance (both common conditions in renal I/RI). However, exogenous administration of Ac2-26 has been shown to reverse these side effects [77].

Diabetes is metabolic syndrome which affects many organs including the kidneys, with 30–40% of diabetic patients developing nephropathy [78]. AnxA1 has been shown to afford protection in murine diabetic nephropathy by decreasing p38, ERK and JNK, and activating Akt signaling, suggesting that AnxA1 could be a potential therapeutic strategy for treating renal dysfunction caused by diseases such as diabetes [79].

## 7. I/RI in Gastrointestinal (GI) System

I/RI in the GI system is mainly seen with abdominal and thoracic vascular surgery, but it can also be associated with small bowel transplantation and hemorrhagic shock [80]. Most of these GI complications lead to mesenteric ischemia and I/RI. The mortality rate associated with mesenteric I/RI is in the range of 40–60% [81]. Enterocytes are the main intestinal cells and are resistant to short term hypoxia, however prolonged hypoxia can cause irreversible cell death resulting in the production of various DAMPs, e.g., nucleic acids and heat shock proteins. Ischemia causes inactivation of enzymes such as cytochrome oxidase and superoxide dismutase, so upon re-oxygenation, oxidative phosphorylation does not occur, resulting in ROS production which can further trigger apoptosis of enterocytes further causing intestinal barrier disruption. There is also increase in intracellular calcium concentration leading to changes in cytoskeleton, which further damages cell–cell junctions, thereby increasing vascular permeability [82].

Systemic inflammation is a significant component of intestinal I/RI. The loss of barrier function facilitates movement of microbiota and their products from the gut to the blood stream. The molecular patterns associated with these microbes and their products, known as pathogen-associated molecules pattern (PAMPs). PAMPS and DAMPs can bind TLRs on immune cells, activating local inflammatory response such as production of cytokines, cell adhesion molecules, and chemokine receptors via induction of NF-κB. These steps lead to recruitment and infiltration of leukocytes causing tissue damage [82]. The inflamed intestinal mucosa also produces as whole plethora of different inflammatory cytokines and chemokines, e.g., TNF-α, IL-12, IL-15 and IL-23, which can significantly increase leukocyte infiltration and exacerbate inflammation and disease [83].

### The Protective Role of AnxA1 in GI-Associated I/RI

AnxA1 has been shown to play a protective role in gastrointestinal I/RI. We have shown that intravenous administration of Ac2-26 inhibits leukocyte adhesion and emigration in a mouse mesenteric I/RI model, as induced by clamping of the superior mesenteric artery, followed by reperfusion [84]. The loss of epithelial cell barrier is major characteristic of intestinal I/RI. The effect of AnxA1 or its peptides on epithelial cell barrier has not been studied in intestinal I/RI models but they have afforded protection in other gastrointestinal inflammatory murine models including colitis and gastric ulcers. Leoni et al. demonstrated that Ac2-26, when delivered locally in the intestine using polymeric nanoparticles, enhanced the epithelial cell restoration in colitis [85]. Martin et al. showed the positive effect of peptide Ac2-26 in enhancing the healing process of gastric mucosal damage and reducing the ulcer areas. Furthermore, this same group also demonstrated that hemorrhagic lesions were greater in AnxA1^−/−^ mice upon induction of gastric ulcers [86]. Leoni et al. suggested that AnxA1 exhibits an FPR1/NOX1-dependent positive role in intestinal wound healing: upon binding to FPR1, AnxA1 activates Src and downstream NOX-1 [87]. ROS produced by NOX-1 causes rapid and reversible oxidation and subsequent inactivation of phosphatase PTEN and PTP-PEST. This inactivation of phosphatases leads to activation of focal adhesion protein involved in cell movement, FAX and Paxillin, thereby increasing the intestinal epithelial cell movement and wound repair [87].

Intestinal I/RI is also a major cause of acute lung injury (ALI). Treatment with exogenous Ac2-26 has been shown to reduce pulmonary vascular leakage, decrease neutrophil infiltration and MPO content in lung tissues following intestinal I/RI, possibly by inducing release of anti-inflammatory cytokine IL-10 and decreasing the release of pro-inflammatory cytokine TNF-α [88]. Babbin et al. also showed in a dextran-sulfate sodium (DSS)-induced colitis model that AnxA1 was able to regulate intestinal mucosal injury and inflammation via engagement with FPR2/ALX. In addition, AnxA1 deficient mice failed to regain weight and showed no improvement in disease activity index and mucosal injury upon withdrawal of disease in the DSS-induced colitis model [89], advocating the crucial and beneficial role of AnxA1.

## 8. Concluding Remarks

**Figure 1:**
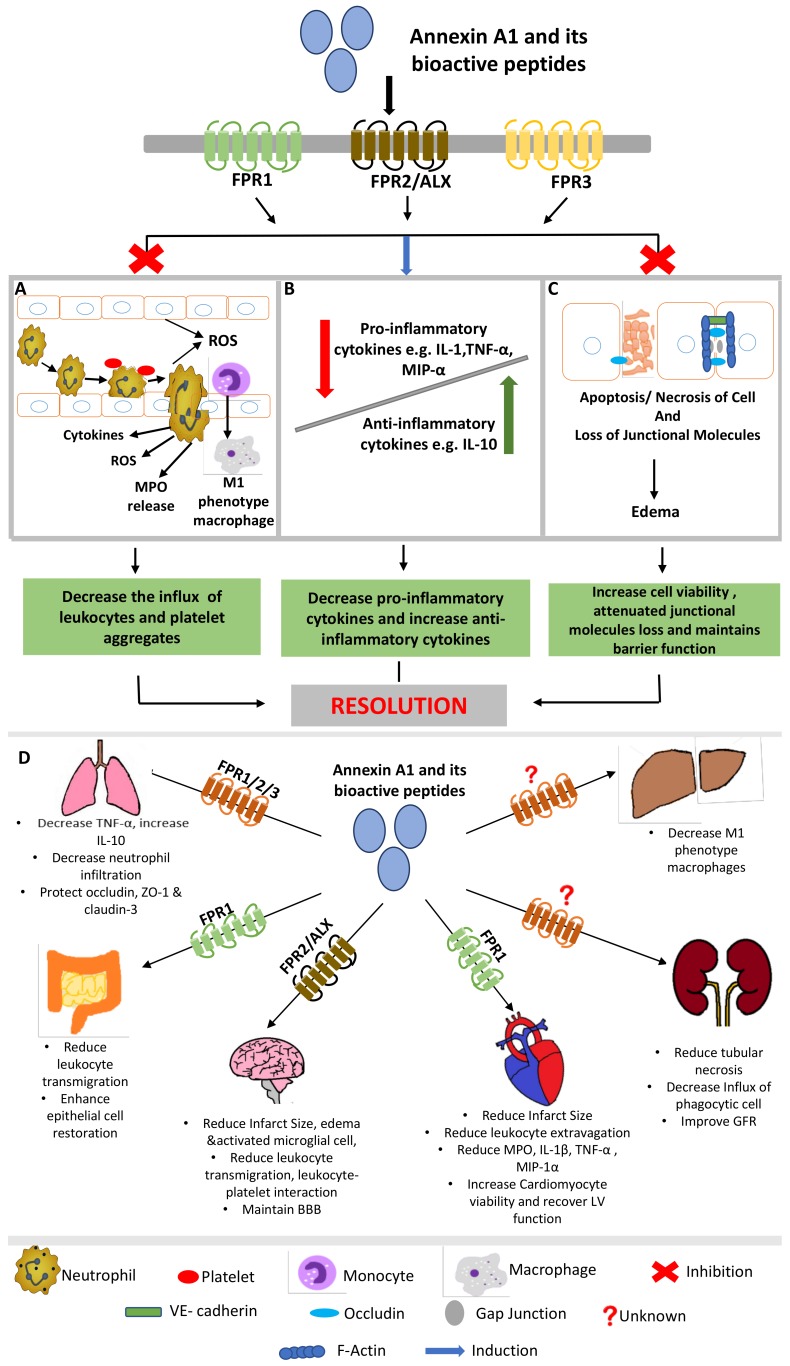
Schematic representation of the protective effects of AnxA1 in I/RI. By binding to members of the FPR family, AnxA1 and its peptide mimetics can (**A**) decrease rolling, adhesion and emigration of leukocytes (monocytes and neutrophils) [31,46,47,53,76,84,88]. In addition, platelet aggregates are diminished [7,31,47] and pro-inflammatory mediators (e.g., myeloperoxidase (MPO), reactive oxygen species (ROS) and cytokines) are moderated [88,90]. (**B**) AnxA1 promotes the balance between pro-inflammatory [7,31,47,58,60,88,90] and anti-inflammatory cytokines, thereby fostering homeostasis in the host. (**C**) The disruption of barrier function, due to the loss of junctional molecules and apoptosis/necrosis of endothelial/epithelial cells, is one of the main characteristics of I/RI. AnxA1 has a positive effect on maintaining barrier function [49] and reducing edema [48] by inhibiting the loss of junction molecules, in particular occludin, VE-cadherin and actin cytoskeleton [50,59]. Thus, AnxA1 regulates the inflammatory responses elicited during I/RI leading to the resolution of inflammation and decreased tissue injury in (**D**) different vascular beds.

In summary, CVDs are the leading cause of morbidity and mortality worldwide, with MI and IS being the primary causes of death. The majority of CVD related complications are due to I/RI. Hence, it has become imperative to develop preventative as well as therapeutic strategies to mitigate these life-threatening complications. In this review, we focus on AnxA1 and its peptide mimetics as possible therapeutic strategies for the treatment of I/RI, based on their ability to alleviate thrombosis and inflammation and promote resolution (Figure 1). Indeed, drug development programs focused on endogenous anti-inflammatory and pro-resolving agents, such as AnxA1-based pharmacologic strategies, offer great therapeutic potential as they are devoid of metabolic side effects because they mimic the way inflammation naturally subsides in the body. However, the development of novel therapeutics should also be mindful of ascertaining not only if the drug is anti-inflammatory, but also if it is resolution-toxic (i.e., deranges or impairs timely and/or complete resolution), which could ultimately outweigh the overall therapeutic efficacy in treating I/RI and other inflammatory disorders. 

## Figures and Tables

**Table 1:** FPR nomenclature and cellular and tissue distribution in human and mouse.

Species	IUPHAR Nomenclature	Cellular Distribution	Tissue Distribution
HUMAN	FPR1	Adrenal cortical cells, astrocytes, carcinoma cells, endothelial cell, epithelial cells, fibroblasts, kupffer cells, macrophages, microglial cells, monocytes, neuroblastoma cells, neutrophil, platelets, immature dendritic cells, hepatocytes	Adrenal glands, bone marrow, central nervous system, colon, eye, heart, kidney, liver, lung, ovary, placenta, spleen
	FPR2/ALX	Astrocytes, endothelial cells, epithelial cells, fibroblasts, hepatocytes, immature dendritic cells, microglial cells, macrophages, monocytes, neuroblastoma cells, neutrophils, T and B lymphocytes,	Bone marrow, brain, lung, placenta, spleen, testis
	FPR3	Dendritic cells, HL-60 cells, macrophages, monocytes, eosinophils	Adrenal gland, liver, lung, lymph nodes, placenta, small intestine, spleen, trachea
MURINE	Fpr1	Dendritic cells, microglia, mononuclear cells, neutrophils	Adrenal gland, anterior pituitary, hippocampus, hypothalamus, liver, lung, spleen
	Fpr2	Dendritic cells, microglia, neutrophils	Anterior pituitary, adrenal gland, hippocampus, hypothalamus, lungs, spleen
	Fpr3	Microglia, neutrophils	Adrenal gland, anterior pituitary, heart, hippocampus, hypothalamus, liver, lung, spleen
	Fpr-rs3		Skeletal muscle
	Fpr-rs4		unknown
	Fpr-rs5		unknown
	Fpr-rs6		Brain, skeletal muscle, spleen, testis
	Fpr-rs7	Smooth muscle	Heart, liver, lung, pancreas, spleen, smooth muscle
	Fpr-rs8		unknown

**Table 2:** Non-exhaustive list of FPR ligands and their biological actions.

Ligand	Biological Action	Disease State	Refs
**FPR agonists**			
Aβ_42_ (FPR2/ALX)	Chemotaxis of mononuclear cells	Bacterial pathogenesis	[90]
Ac2-12 (FPR1)	Cardioprotection in experimental MI/R	MI [58]	[58]
Ac2-26 (FPR1, FPR2/ALX)	Decreases neutrophil–endothelium interactions in flow chamber Regulates leukocyte–platelet response in the cerebral microvasculature Decreases pulmonary arterial pressure Decreases inflammatory cytokine production Decreases lung tissue damage following I/R Decreases infarct size post myocardial I/R Reduces myeloperoxidase activity Prevents IFN-γ and endotoxin induced inotropic and cyclooxygenase 2 gene expression Decreases adhesion and transmigration in inflammatory mesentery in vivo	AIS [31] I/R induced lung injury [91] MI [33,58] Mesenteric ischemia [84]	[31,33,58,84,91]
Ac9–25 (FPR1)	Activates neutrophil NADPH oxidase	Inflammation	[92]
Annexin A1 (FPR2/ALX)	Regulates microglial efferocytosis and phagocytosis Decreases neutrophil–endothelium interactions in flow chamber Cardioprotection in experimental MI/R Prevents leukocyte migration to the inflamed tissue	AIS [93] MI [58]	[58,93]
Antiflammin 2 (FPR2/ALX)	Decreases neutrophil–endothelium interactions	Inflammation	[22]
Cathepsin G (FPR1)	Chemoattractant for phagocytic leukocytes Promotes platelet aggregation and hemostasis Promotes thrombus formation Promotes middle cerebral artery occlusion and brain injury in ischemic stroke model	AIS [94]	[94,95]
CRAMP (FPR2/ALX)	Chemotactic activator of mouse and human leukocytes Calcium flux, MAPK activation	Bacterial pathogenesis	[96]
Compound 17b (FPR1, FPR2/ALX)	Attenuates early as well as late inflammatory responses after via ERK1/2–Akt kinase system	MI [64]	[64]
D2D3_88–274_ (FPR2/ALX)	Inhibits monocyte chemotaxis and integrin-dependent cell adhesion	Inflammation	[97]
fMLP and analogues (FPR2/ALX)	Defective PMN chemotaxis in juvenile peridontitis in vivo Lineage specific differentiation of mesenchymal stem cells especially osteoblasts	Juvenile periodontitis [98] Osteoporosis [99]	[98,99]
Formylated humanin (FPR2/ALX)	Chemotaxis of human FPR2/ALX-transfected CHO cells	Inflammation	[100]
Humanin (FPR2/ALX)	Chemotaxis of human FPR2/ALX-transfected CHO cells	Inflammation	[100]
HIV-1 T20 (DP178) (FPR1, FPR2/ALX)	Chemoattractant and activator of peripheral phagocytes, hence promoting host immune responses against HIV-1 replication	HIV/AIDS [101,102]	[101,102]
HIV-1 T21 (DP107) (FPR1, FPR2/ALX)	Chemoattractant and activator of peripheral phagocytes (high affinity towards FPR2/ALX)	HIV/AIDS [103]	[103]
HIV gp41 (N36) (FPR2/ALX)	Induces directional migration and calcium mobilization in human monocytes and neutrophils	HIV/AIDS [104]	[104]
Lipoxin A_4_ and ATL (aspirin triggered lipoxin)	Inhibition of lung inflammation after hind-limb IR Downregulation of neutrophil accumulation Regulates neutrophil–platelet aggregates ATL is required for ASA protection in AIS	Hind limb ischemia [105] AIS [31]	[31,105]
LL-37 (cathelicidin peptide) (FPR2/ALX)	Enhances phagocytosis of IgG-opsonized Gram negative and Gram-positive bacteria Chemoattractant for human peripheral blood neutrophils, monocytes and T cells Calcium mobilization	Bacterial pathogenesis	[106]
MMK-1 (FPR2/ALX)	Potent chemoattractant and calcium mobilizing agent agonist for human monocytes, neutrophils and FPR2/ALX transfected human embryonic kidney (HEK) 293 cells.	Bacterial pathogenesis	[107]
N36 peptide (FPR2/ALX)	Chemotaxis and calcium mobilization in monocytes and neutrophils	Viral pathogenesis	[104]
NADH dehydrogenase (FPR2/ALX)	Chemotaxis and calcium mobilization in human FPR2/ALX-expressing HL-60 cells	Inflammation	[108]
PACAP27 (FPR2/ALX)	Neutrophil chemotaxis and upregulation of CD11b Intracellular calcium mobilization ERK phosphorylation	Inflammation	[109]
PRP106-126 (FPR2/ALX)	Endocytosis in glial cells	Neurodegenerative diseases	[110]
Quin-C1 (FPR2/ALX)	Neutrophil chemotaxis, stimulates calcium mobilization, and MAP kinase phosphorylation	Inflammation	[111]
Rana-6 (FPR2/ALX)	Chemoattractant of phagocytes	Inflammation	[112]
SRSRY (FPR1)	Directional cell migration on vitronectin-coated filters	Inflammation	[113]
Serum amyloid A (SAA) (FPR2/ALX)	Potent leukocyte chemoattractant	Inflammation	[114]
Temporin A (FPR2/ALX)	Chemoattractant and activator of peripheral phagocytes	Bacterial pathogenesis	[96]
uPAR_84–95_ (FPR2/ALX)	Chemoattractant and activator of peripheral phagocytes (high affinity)	Inflammmation	[115]
V3 peptide	Chemoattractant of phagocytes Inhibits monocytic response to chemokines	Inflammation	[115]
W peptide (FPR2/ALX)	Activates phagocyte chemotaxis and calcium flux	Inflammation	[116]
**FPR antagonists**			
BOC2 (FPR1, FPR2/ALX)	Decreased neutrophil activation	Inflammation	[117]
CDCA (FPR1)	Inhibits neutrophil chemoattraction and migration Inhibits calcium flux	Leukocyte migration/Inflammation	[118]
CHIPS (FPR1)	Inhibits chemotaxis in *S. aureus* infection	Bacterial pathogenesis	[119]
Coronavirus 229E peptides (FPR2/ALX)	Ligand binding studies using transfected CHO cells demonstrated antagonism of FPR2/ALX	Viral/bacterial pathogenesis	[120]
Coronavirus peptides (FPR2/ALX)	Inhibits fMLP interaction in CHO cells	Viral/bacterial pathogenesis	[120]
Cyclosporine A (FPR1)	Inhibits fMLF-stimulated degranulation, chemotaxis, calcium mobilization of neutrophils	Inflammation	[121]
Cyclosporine H (FPR1)	Decreased neutrophil activation	Inflammation	[117]
DCA (FPR1)	Inhibits fMLP-induced monocyte and neutrophil chemotaxis and calcium mobilization	Inflammation	[112]
Ebola peptides (FPR1)	Inhibits fMLP interaction in CHO cells	Viral pathogenesis	[120]
FLIPr (FPR2/ALX)	FPR2/ALX inhibitory protein (FLIPr) exerts anti-inflammatory activity by inhibiting calcium mobilization and cell migration toward chemoattractants.	Inflammation	[122]
HIV-2 peptides (FPR1)	Inhibits fMLP interaction in CHO cells	Viral pathogenesis	[120]
Isopropylureido-FLFLF (FPR1)	Inhibits chemotaxis	Inflammation	[123]
Spinorphin (FPR1)	Inhibits calcium mobilization and fMLP induced neutrophil chemotaxis	Inflammation	[124]
WRW4 (FPR2/ALX)	Inhibits chemotaxis, calcium flux, superoxide generation and ERK phosphorylation	Neurodegenerative diseases, AIS	[125]

MI, myocardial infarction; MI/R, myocardial ischemia reperfusion; AIS, acute ischemic stroke; IFN-γ, interferon gamma; MAPK, Mitogen-activated protein kinase; ERK, extracellular signal–regulated kinase; AKT, serine/threonine-protein kinase; PMN, polymorphonuclear leukocytes; CHO cells, Chinese hamster ovary; ATL, aspirin triggered lipoxin; ASA, aspirin ; IgG, Immunoglobulin G; fMLP, formyl-Met-Leu-Phe (fMLP), G proteins; FLIPr, FPR2/ALX inhibitory protein
